# Validation of the Behavioral Anger Response Questionnaire for Children (BARQ-C) in a large community sample of Vietnamese middle adolescents in Hanoi

**DOI:** 10.1186/s40359-022-00907-4

**Published:** 2022-08-12

**Authors:** Ian Shochet, Jayne Orr, Wendell Cockshaw, Thach Tran, Nga La, Huong Nguyen, Nga Nguyen, Astrid Wurfl, Hau Nguyen, Ruby Stocker, Jane Fisher

**Affiliations:** 1 School of Psychology and Counselling, Queensland University of Technology, Kelvin Grove, Brisbane, QLD 4059 Australia; 2 The Australian Centre for Health Services Innovation, Queensland University of Technology, Brisbane, QLD Australia; 3 Global and Women’s Health, Public Health and Preventive Medicine, Monash University, Melbourne, VIC Australia; 4 Hanoi University of Public Health, Hanoi, Vietnam

**Keywords:** Construct validity, Anger coping, Depression detection and prevention, Detection and prevention of mental health difficulties, Adolescents, Vietnam, BARQ-C

## Abstract

**Background:**

Depression and other forms of psychological distress are common among Vietnamese adolescents and increase the risk of mental health problems in adulthood. As anger coping is a robust predictor of adolescent mental health difficulties, and there appear to be cultural variations in anger coping, a measure of adolescent anger coping styles that has been validated using a non-Western adolescent sample is required to inform and support early intervention to prevent or treat mental health difficulties in Vietnamese adolescents. This study examined the construct validity (structural and external) of the Behavioral Anger Response Questionnaire for Children in Vietnam (BARQC-V).

**Methods:**

Baseline data sourced from a recent randomised control trial conducted with Grade 10 Vietnamese adolescents aged 14 to 16 (*N* = 1084) were used to examine multiple aspects of construct validity: factorial structure (evaluated using factor analysis); internal consistency (tested using Cronbach’s alpha coefficient); and external aspect (assessed using Pearson’s correlation coefficients between the BARQC-V and Vietnamese translations of the Coping Self-Efficacy Scale, Centre for Epidemiologic Studies Depression Scale Revised, Mental Health Continuum Short Form, and the Depression Anxiety and Stress Scale).

**Results:**

Evaluating factorial structure using confirmatory factor analysis failed to converge on a solution. Exploratory factor analysis yielded a 5-factor structure model that explained 49.32% of the BARQC-V’s total variance and was deemed to be a good fit by the final confirmatory factor analysis. Cronbach’s alpha coefficients of the 5 factors demonstrated acceptable internal reliability for the BARQC-V’s sub-scales. Concerning concurrent validity, three sub-scales predicted well-being and mental health difficulties: the maladaptive anger coping styles Rumination and Direct Anger-out were positively associated with depression and distress, and negatively associated with coping self-efficacy and mental well-being; and the adaptive anger coping style Assertion was positively associated with coping self-efficacy and mental well-being, and negatively associated with depression.

**Conclusions:**

The BARQC-V provides a validated measure of three anger coping strategies used by adolescents in Vietnam (Rumination, Direct Anger-out, and Assertion) that can be used to improve detection and treatment of mental health difficulties in this population, and as a starting point by future research to develop a much-needed gold standard measure of anger coping for adults, adolescents and children world-wide.

## Background

Depression and other forms of psychological distress are common among Vietnamese adolescents and increase the risk of mental health problems in adulthood [1, 2]. Anger coping, the modification of heightened anger by aggressively venting or keeping angry feelings to oneself [3], is a robust predictor of mental health difficulties in adolescents. and there are cultural variations in anger coping [4]. To detect maladaptive anger coping in Vietnamese adolescents to facilitate intervention to prevent and diminish psychological distress, a measure of adolescent anger coping styles validated using a non-Western population is required.

Anger, a negative feeling state with specific cognitive appraisals, physiological changes and action tendencies [5], is associated with several mental disorders, many of which do not include anger as a key symptom, for example mood disorders, disrupted eating, and substance-related disorders [6, 7]. Research shows a close relationship between anger and depression in community [8, 9] and patient populations [10, 11], with depressed people experiencing more anger [12] and using more anger suppression (concealment of angry feelings) than non-depressed people [9]. Although anger is generally perceived as maladaptive [13, 14], it can be adaptive (i.e., protective against psychopathology), especially when it is communicated in a helpful manner [15]. As adolescents commonly experience anger, which has been linked with mental health problems during puberty [16, 17], and has been found to predict maladjustment in adulthood [7, 18, 19], understanding processes used to manage anger is important.

### Anger coping

Emotion regulation refers to cognitive strategies used to manage feelings, thoughts and behaviour to cope with challenging situations in an effective and socially acceptable manner [20, 21]. Helpful (adaptive) strategies are associated with mental health, while unhelpful (maladaptive) strategies are associated with mental illness [22, 23].

Three anger coping emotion-regulation strategies are theorised to be adaptive: reappraisal, the generation of benign or positive interpretations of a stressful situation to reduce distress [24, 25]; problem solving, a conscious attempt to change a stressful situation or contain its consequences [26, 27]; and acceptance of an experienced emotion without attempting to control or change it [25, 28]. Conversely, three maladaptive anger coping emotion-regulation strategies increase the risk of depression: suppression of unwanted thoughts which paradoxically increases the accessibility of the suppressed thought, increases emotional arousal, and interferes with habituation to emotional stimuli [29, 30]; avoidance which increases negative thoughts and prevents remedial action [30]; and rumination which interferes with effective problem solving and increases indecision [31] by repetitively focusing on the emotion and its causes and consequences [32, 33]. A meta-analysis of 114 studies examined the relationships between these six emotion-regulation strategies and symptoms of internalising disorders (anxiety and depression) and externalising disorders (disrupted eating and substance-related disorders) [6]. Acceptance, reappraisal and problem solving were associated with reduced anxiety and depression, while rumination, avoidance and suppression were associated with increased anxiety and depression. Effect sizes (interpreted as large (> 0.40), medium (0.10–0.39), or small (< 0.10) [34]) emerged as large for rumination; medium to large for avoidance, problem solving, and suppression; and small to medium for reappraisal and acceptance.

Research into cultural variations in anger coping is in its infancy [4]. It has been postulated that Asian cultures tend to value family and the group ahead of individual needs; emphasise harmony, self-restraint and affective control; and encourage individuals to adapt to their environment in order to fit in [35, 36]. Anger tends to be socially perceived as maladaptive for relationships and is strongly discouraged as its expression may draw attention to the individual, encourage disharmony, and demonstrate a lack of self-restraint [37, 38]. Studies with Vietnamese children and adults living in America suggest that they are more likely than their Western counterparts to suppress anger [39, 40], implying that Vietnamese adolescents living in Vietnam would suppress their anger also. However, the manner in which Vietnamese adolescents living in Vietnam actually cope with anger is unknown.

### Measuring anger coping

Although many anger coping styles likely exist [41–43], a preponderance of anger coping scales only measure a few anger coping styles such as anger-out (expressed anger) or anger-in (suppressed anger). The Behavioral Anger Response Questionnaire (BARQ) was developed to measure anger coping in adults using 37 items grouped into six sub-scales, each of which represented an anger coping style [44]. It was suggested that two sub-scales represented maladaptive anger coping styles: Direct Anger-out (extreme expression) and Avoidance (extreme suppression and similar to anger-in); that three sub-scales represented adaptive anger coping styles: Assertion (akin to problem-solving), Social Support-seeking (recruiting support from others to boost anger coping), and Diffusion (suppressing or redirecting angry feelings e.g., by engaging in another activity); and that Rumination measured the degree to which adults repeatedly think about their response to anger [44]. The BARQ has demonstrated good internal consistency (mean α = 0.76, range 0.65–0.85) and acceptable construct validity [44].

Martin and Dahlen [42] compared the BARQ to the State-Trait Anger Expression Inventory-2 (STAXI-2) [45]. Findings suggested that Rumination represented extreme suppression, contrary to an earlier suggestion that Avoidance represented extreme suppression; that Direct Anger-out and Rumination were maladaptive anger coping styles; and that Assertion, Social Support-seeking, Diffusion and Avoidance were adaptive anger coping styles. They recommended additional research to re-evaluate the meaning of the BARQ sub-scales.

The Behavioral Anger Response Questionnaire for Children (BARQ-C) was developed to measure anger coping styles in children and adolescents [3] by rewording some items to make them more relevant to this population, and was validated using a sample aged 11.1–16.3 years old (British *n* = 393, Dutch *n* = 299). The Social Support-seeking and Assertion anger coping sub-scales yielded good psychometric properties in both samples. Good internal consistency for Direct Anger-out and Rumination emerged after removing one Direct Anger-out item and two Rumination items that did not load on their intended factors. Cronbach’s alphas for the Diffusion and Avoidance sub-scales were unacceptable but all items were retained as removing weaker items highlighted by factor analysis did not improve internal consistency. The authors recommended that future research endeavour to improve the BARQ-C.

A few studies have used the BARQ-C in populations from Asia. Using the 37-item BARQ-C to explore the influence of anger coping on the mental health and friendship quality of Chinese adolescents (*N* = 630, 12–16 years old) found that the influence of Social Support-seeking on mental health problems was fully mediated by friendship quality, the influence of Assertion on mental health problems was partially mediated by friendship quality, and mental health problems were predicted by the Direct Anger-out and Rumination coping styles [46]. The impact of self-esteem (measured using the Rosenberg Self-esteem Scale) and anger coping (measured using a 33-item version of the BARQ-C after removing one Rumination item, one Avoidance item and two Diffusion items) on anger was explored with adolescents in Indonesia with a history of juvenile delinquency (*N* = 178, 12–20 years old) [47]. Direct Anger-out and Rumination had a significant influence on the level of anger. A modified 24-item BARQ-C was used to explore the socio-cultural perspective on anger regulation in Chinese children from Hong Kong and Dutch Children (*N* = 131, 11 years old) [48]. Chinese children were more likely to react tolerantly to the aggressor, whereas Dutch children indicated that they would verbally confront the aggressor. Although the BARQ-C has not been validated using a non-Western population, given the lack of a gold standard measure of anger coping (in adults and/or children regardless of culture), face validity of the BARQ-C suggested that it had potential for research in Vietnam to detect anger coping among adolescents.

### Anger and mental health of adolescents in Vietnam

Depression and other mental health problems in adolescence are a prominent public health problem worldwide [49], with depressive disorders the fourth leading cause of disability-adjusted life-years in adolescents aged 10–24 years [50]. As the majority of first onset depression occurs during adolescence [51] and increases the risk of mental health problems in adulthood [52], adolescence offers a crucial window for the prevention, detection and early treatment of depression. Mental health problems, including depression, are common among adolescents in Vietnam, with up to 23% of Vietnamese adolescents experiencing clinically significant symptoms of depression [53]. While findings show that anger coping styles predict mental health problems in adolescents, and continue to impact on mental health in adulthood [54], there is a paucity of research exploring this association in non-Western cultures. Hence, a validated instrument that measures anger coping styles used by Vietnamese adolescents is required to inform and support early intervention to prevent or treat depression.

### The present study

This study aimed to examine multiple aspects of the construct validity of the BARQ-C for use with high school students in Vietnam, including its structural aspect (factorial structure, measurement invariance, and internal consistency), and external aspect (concurrent validity). The study used baseline data collected in a school-based cluster randomised controlled trial (RCT) that explored the impact of a resilience intervention on the mental health of Vietnamese adolescents in Hanoi [55]. Considering past research, it was hypothesised that the Direct Anger-out and Rumination anger coping styles would be maladaptive, and therefore positively associated with depression and distress, and negatively associated with coping self-efficacy and mental well-being [46, 47]. Further, it was hypothesised that the Assertion, Social-support Seeking, Avoidance and Diffusion anger coping styles would be adaptive, and therefore positively associated with coping self-efficacy and mental well-being, and negatively associated with depression and distress [24–28].

## Methods

### Setting

The study took place in Hanoi, the capital of Vietnam. Vietnam is a Southeast Asian country with a population of 96 million, 8 million of whom live in Hanoi. Children and adolescents account for a third of Vietnam’s population, and about 92% of school-age children (6–18 years old) attend school [56]. Hanoi’s population is split equally between those living in its urban and rural districts. The average national per capita income in 2019 was USD2,590, and Vietnam is classified as a lower-middle income country [57].

### Study design and participants

This is a secondary analysis of baseline data collected using a multiple-stage sampling method in the aforementioned RCT of adolescent mental health in Vietnam [55]. An independent statistician conducted the selection process. In the first stage, two districts were randomly selected from 12 urban districts and another two districts were randomly selected from 18 rural districts in Hanoi. In the second stage, in each of the selected districts, two high schools were randomly selected and four grade 10 classes from each of the selected schools were randomly chosen. All students in the selected classes (14–16 years old) were invited to participate in the RCT, with 1084 students providing consent (552 controls and 532 interventions; 60.7% female, *M*_*age*_ = 15.3 years). All participants of the RCT were included in this validation study.

### Measures

All measures were translated into Vietnamese using a standardised procedure (translate, culturally verify and back-translate). This procedure has been established and used in previous studies [58–60].

#### Behavioral Anger Response Questionnaire for Children and Adolescents (BARQ-C) [3]

Anger coping strategies were assessed using the 37-item BARQ-C translated into Vietnamese (BARQC-V). The BARQ-C includes statements about the manner in which the respondent might react when feeling angry, e.g., “I put what happened out of my mind”, “I hit or push the person who made me angry”, and “In a calm voice, I tell the person who made me angry how I honestly feel”. Each statement is rated on a three-point scale according to the extent to which the respondent endorses the statement: 1 (not true), 2 (sometimes true), or 3 (often true). The items are typically grouped into six anger coping strategies (sub-scales): Direct Anger-out (7 items); Assertion (6 items); Social Support-seeking (6 items); Diffusion (6 items); Avoidance (6 items); and Rumination (6 items). Item scores within each sub-scale are summed to obtain a score for each sub-scale, with higher sub-scale scores indicating that the respondent is more likely to use the anger coping strategy.

#### Coping Self-Efficacy Scale (CSES) [61]

The CSES is a 26-item measure of one’s confidence in performing coping behaviours when faced with life challenges and demonstrates high reliability (α = 0.95). Using the CSES translated into Vietnamese (CSES-V), participants responded to item statements (e.g., “Keep from feeling sad”, “Make new friends”, and “Pray or meditate”) on an 11-point Likert scale, ranging from 0 (cannot do at all) to 10 (certain can do). Scores were summed, with higher results showing a greater degree of self-efficacy to cope under duress. Using the baseline data from this RCT, evidence for a 3-factor structure and good internal consistency of the CSES-V for use among Vietnamese adolescents has been established (emotion-focused sub-scale α = 0.91, problem-focused sub-scale α = 0.86, social support/interaction coping strategies sub-scale α = 0.75, whole scale α = 0.93) [62].

#### Centre for Epidemiologic Studies Depression Scale Revised (CESD-R) [63]

Depressive symptoms were measured using the Vietnamese translation of this 20-item scale (CESDR-V) which reflects the Diagnostic and Statistical Manual of Mental Disorders, Fourth Edition (DSM-IV) definition of Major Depressive Disorder [64]. Participants responded to each item (e.g., “I felt sad”, “I slept much more than usual”, and “I wanted to hurt myself”) using a 5-point Likert scale, ranging from 0 (not at all or less than one day in the past week) to 4 (nearly every day for two weeks). The total scale scores ranged from 0 to 80, with higher scores indicating more depressive symptoms. As specified by the developers of this scale, a total score ≥ 16 was indicative of clinically significant depressive symptoms. Construct validity of the CESDR-V using the RCT’s baseline data has yielded evidence of unidimensional measurement, excellent internal consistency (α = 0.92), and measurement invariance between boys and girls.

#### Mental Health Continuum Short Form (MHC-SF) [65]

General mental well-being was assessed using the Vietnamese translation of the 14-item MHC-SF (MHCSF-V). Participants indicated how often over the past month they felt each mental well-being statement e.g., “interested in life”, “confident to think or express your own ideas and opinions”, and “that your life has a sense of direction or meaning to it” using a score from 0 (never) to 5 (every day). Item scores are summed to yield a global well-being score from 0 to 70, with higher global well-being scores indicative of better mental well-being. The construct validity of the MHCSF-V for use in adolescents in Vietnam has been established, and internal consistency was high (α = 0.88) [66].

#### Depression Anxiety and Stress Scales (DASS) [67]

Symptoms of depression, anxiety and stress were assessed using the Vietnamese translation of the 21-item version of the DASS which consists of three 7-item sub-scales that can be summed to yield scores for Depression (DASS21D-V), Anxiety (DASS21A-V) and Stress (DASS21S-V); and a total distress score DASS21-V. Based on their experience over the past week, participants responded to each item (e.g., “I felt that I had nothing to look forward to”, “I felt I was close to panic”, and “I felt that I was using a lot of nervous energy”) using a 4-point Likert scale, ranging from 0 (did not apply to me at all) to 3 (applied to me very much, or most of the time). Higher sub-scale scores indicate more symptoms of the mental health difficulty measured by the sub-scale. Evidence for the factorial structure and internal consistency of the DASS21-V for use among Vietnamese adolescents has been established (DASS21D-V α = 0.84, DASS21A-V α = 0.74, DASS21S-V α = 0.76) [68].

### Procedure

The data analysed in this study were collected at recruitment (baseline) in the RCT (other data collection points were post-intervention (about two months after recruitment), and at 6-month follow-up). An anonymous questionnaire was distributed to the student participants at school during a usual 45-min class. Two research assistants from the Hanoi University of Public Health provided instructions about questionnaire completion and supervised the students to ensure privacy and confidentiality. Students returned the questionnaire in a sealed envelope provided at the beginning of the session. Students who declined to participate or did not have parental consent to participate were invited to do their homework in the school library (44 students, 3.9%).

### Data analysis

This study examined two aspects of construct validity of the BARQC-V: its structural validity (factorial structure, measurement invariance, and internal consistency), and external validity (concurrent validity) [68]. Confirmatory factor analysis was conducted using IMB AMOS version 28 and remaining analyses were carried out using IBM SPSS Statistics 28.0.1.

#### Structural validity

*The factorial structure* of the BARQC-V was examined first using confirmatory factor analysis with 6 correlated factors specified. Next, exploratory factor analysis using maximum likelihood extraction was used. The number of factors selected was decided based on the Kaiser criterion (eigenvalues > 1). The scree plot, total percent variance explained, and meaningful factors were used to select factors if more than one factor met the Kaiser criterion. After the number of factors was determined, an oblique rotation (promax) was applied when more than one factor was found. Items with factor loadings < 0.3 were interpreted as being not salient [69], and were omitted from the final version of the BARQC-V. The *internal consistency* of each BARQC-V sub-scale was tested using Cronbach’s alpha coefficient. A coefficient > 0.7 indicated acceptable internal reliability.

#### External validity

To determine whether the BARQC-V correlated with measures of related constructs, its *concurrent validity* was examined using Pearson’s correlation coefficients between the scores of the three sub-scales of the CSES-V, the CESDR-V, the MHCSF-V, and the three sub-scales of the DASS21-V. Correlations were interpreted as strong (≥ 0.5), moderate (0.3–0.49), weak (0.1–0.29), or negligible/no correlation (< 0.1) [34].

#### Data screening

All BARQC-V items exhibited a spread of responses. The 3-point response scale limits the assessment of normality. However, the central response was the most common for all but nine items. There were little missing data, with less than 1% missing for all items (maximum *n* = 7).

## Results

### Structural validity

#### Factorial structure

An initial confirmatory factor analysis using the 37 BARQC-V items with 6 correlated factors specified failed to converge on a solution due to multiple mis-specified items (see Table 1). Exploratory factor analysis specifying 6 factors and using the maximum likelihood extraction method and promax rotation with Kaiser normalization provided further evidence that a 6-factor model resulted in a poor fit (see Table 2). Using exploratory factor analysis to identify low-loading items by analysing one sub-scale at a time and removing the low-loading items, and then analysing all remaining items and removing those that cross-loaded resulted in a clean 22-item 5-factor solution (see Table 3). While four of the five factors are made up of a subset of items within their original factor, one factor is a combination of Diffusion and Avoidance items and has been renamed “Distraction”. The scree plot confirmed five eigenvalues above the inflection point (see Table 4). The 5-factor structure explained 49.32% of the total variance of the BARQC-V.

**Table 1:** Initial confirmatory factor analysis factor loadings using 37 BARQC-V items with 6 correlated factors

Item		Original sub-scale	Factor loading
1	I wait until I am calm again and then talk to the person who made me angry	Assertion	.446
2	In an angry way I tell the person who made me angry exactly how I feel	Direct anger-out	.497
3	I do not show my anger but I talk about what happened with someone afterwards	Social support-seeking	.420
4	I try to understand why I got upset	Rumination	.101
5	I tell myself that what happened is not important	Avoidance	.239
6	I get rid of my anger by playing music, writing, or painting	Diffusion	.392
7	I carefully think it over and then tell the person who made me angry how I feel	Assertion	.628
8	I say something nasty to the person who made me angry	Direct anger-out	.576
9	I leave the situation and look for someone who will agree with me	Social support-seeking	.496
10	I imagine how I could get even with the person who made me angry	Rumination	.441
11	I try to forget what happened	Avoidance	.386
12	I just keep busy, until I stop feeling angry	Diffusion	.454
13	In a calm voice, I tell the person who made me angry how I honestly feel	Assertion	.644
14	I use strong gestures (for example, make a fist, wave my arms, or give a hand sign)	Direct anger-out	.538
15	I leave the situation, find someone to listen to my story, and ask for advice	Social support-seeking	.664
16	I keep thinking about what I wish I had done, but didn’t do	Rumination	.489
17	I put what happened out of my mind	Avoidance	.309
18	I work off my anger by doing some sport	Diffusion	.145
19	I try to understand what happened, so I can explain things to the person who made me angry	Assertion	.566
20	I swear, or curse at the person who made me angry	Direct anger-out	.608
21	I think about the problem first and then talk about it with someone	Social support-seeking	.662
22	I find it hard to stop thinking about what happened	Rumination	.576
23	I do not want to have to cause trouble, so I keep my feelings to myself	Avoidance	.320
24	I stay on my own to get rid of my anger	Diffusion	.451
25	I stay calm, and I try to talk about the problem with the person who made me angry	Assertion	.646
26	I hit or push the person who made me angry	Direct anger-out	.485
27	I leave the situation and call a friend or family member to tell him/her how I feel	Social support-seeking	.539
28	I am upset for a long time after this kind of situation	Rumination	.598
29	I just wait to feel better	Avoidance	.405
30	I simply get very busy with other things to get rid of my anger	Diffusion	.643
31	I leave the situation in order to calm down, and then try to solve the problem	Assertion	.583
32	I express my anger by slamming a door, or hitting something	Direct anger-out	.592
33	Even without planning it, I usually end up talking about my feelings with someone	Social support-seeking	.514
34	In my mind, I go over the situation that made me angry again and again	Rumination	.591
35	I try to keep busy so I can forget about what happened	Avoidance	.577
36	I work off my anger by doing something else, like playing on the computer	Diffusion	.360
37	I shout	Direct anger-out	.396

**Table 2:** Factor loadings from the exploratory factor analysis using 37 BARQC-V items with 6 correlated factors

	Item	Original sub-scale	Factor loading					
			1	2	3	4	5	6
1	I wait until I am calm again and then talk to the person who made me angry	Assertion		.348				
7	I carefully think it over and then tell the person who made me angry how I feel	Assertion		.641				
13	In a calm voice, I tell the person who made me angry how I honestly feel	Assertion		.658				
19	I try to understand what happened, so I can explain things to the person who made me angry	Assertion		.618				
25	I stay calm, and I try to talk about the problem with the person who made me angry	Assertion		.713				
31	I leave the situation in order to calm down, and then try to solve the problem	Assertion		.420				
5	I tell myself that what happened is not important	Avoidance					.313	
11	I try to forget what happened	Avoidance					.511	
17	I put what happened out of my mind	Avoidance					.602	
23	I do not want to have to cause trouble, so I keep my feelings to myself	Avoidance				.469		
29	I just wait to feel better	Avoidance				.342		
35	I try to keep busy so I can forget about what happened	Avoidance						.907
2	In an angry way I tell the person who made me angry exactly how I feel	Direct anger-out	.417					
8	I say something nasty to the person who made me angry	Direct anger-out	.489					
14	I use strong gestures (for example, make a fist, wave my arms, or give a hand sign)	Direct anger-out	.688					
20	I swear, or curse at the person who made me angry	Direct anger-out	.539					
26	I hit or push the person who made me angry	Direct anger-out	.633					
32	I express my anger by slamming a door, or hitting something	Direct anger-out	.510					
37	I shout	Direct anger-out	.357					
6	I get rid of my anger by playing music, writing, or painting	Diffusion					.422	
12	I just keep busy, until I stop feeling angry	Diffusion					.431	
18	I work off my anger by doing some sport	Diffusion				-.312		
24	I stay on my own to get rid of my anger	Diffusion				.530	.313	
30	I simply get very busy with other things to get rid of my anger	Diffusion						.625
36	I work off my anger by doing something else, like playing on the computer	Diffusion						
4	I try to understand why I got upset	Rumination		.368				
10	I imagine how I could get even with the person who made me angry	Rumination	.443					
16	I keep thinking about what I wish I had done, but didn’t do	Rumination				.478		
22	I find it hard to stop thinking about what happened	Rumination				.506		
28	I am upset for a long time after this kind of situation	Rumination				.495		
34	In my mind, I go over the situation that made me angry again and again	Rumination				.501		
3	I do not show my anger but I talk about what happened with someone afterwards	Social support-seeking			.403			
9	I leave the situation and look for someone who will agree with me	Social support-seeking			.486			
15	I leave the situation, find someone to listen to my story, and ask for advice	Social support-seeking			.680			
21	I think about the problem first and then talk about it with someone	Social support-seeking			.564			
27	I leave the situation and call a friend or family member to tell him/her how I feel	Social support-seeking			.641			
33	Even without planning it, I usually end up talking about my feelings with someone	Social support-seeking			.460

**Table 3:** Exploratory factor analysis factor loadings of the 22-item BARQC-V after removing low- and cross-loading items

	Item	Sub-scale	Factor loading				
			1	2	3	4	5
22	I find it hard to stop thinking about what happened	Rumination	.610				
34	In my mind, I go over the situation that made me angry again and again	Rumination	.604				
28	I am upset for a long time after this kind of situation	Rumination	.570				
16	I keep thinking about what I wish I had done, but didn’t do	Rumination	.527				
25	I stay calm, and I try to talk about the problem with the person who made me angry	Assertion		.755			
13	In a calm voice, I tell the person who made me angry how I honestly feel	Assertion		.629			
7	I carefully think it over and then tell the person who made me angry how I feel	Assertion		.590			
19	I try to understand what happened, so I can explain things to the person who made me angry	Assertion		.582			
15	I leave the situation, find someone to listen to my story, and ask for advice	Social support-seeking			.735		
27	I leave the situation and call a friend or family member to tell him/her how I feel	Social support-seeking			.637		
21	I think about the problem first and then talk about it with someone	Social support-seeking			.486		
9	I leave the situation and look for someone who will agree with me	Social support-seeking			.475		
12	I just keep busy, until I stop feeling angry	Diffusion → Distraction				.566	
11	I try to forget what happened	Avoidance → Distraction				.497	
6	I get rid of my anger by playing music, writing, or painting	Diffusion → Distraction				.493	
17	I put what happened out of my mind	Avoidance → Distraction				.442	
36	I work off my anger by doing something else, like playing on the computer	Diffusion → Distraction				.404	
30	I simply get very busy with other things to get rid of my anger	Diffusion → Distraction				.402	
14	I use strong gestures (for example, make a fist, wave my arms, or give a hand sign)	Direct anger-out					.710
26	I hit or push the person who made me angry	Direct anger-out					.673
20	I swear, or curse at the person who made me angry	Direct anger-out					.412
32	I express my anger by slamming a door, or hitting something	Direct anger-out					.393

**Table 4:** Eigenvalues and variance explained of the 22-item BARQC-V

Factor	Eigenvalue	Total variance explained (%)
1. Rumination	3.398	15.45
2. Assertion	3.024	13.75
3. Social Support-seeking	1.727	7.85
4. Distraction	1.435	6.52
5. Direct Anger-out	1.266	5.76

A final confirmatory factor analysis of the modified 5-factor structure converged χ^2^(199) = 762.21. Absolute fit indices indicated acceptable fit, RMSEA = 0.052, 90% CI [0.048, 0.056], SRMR = 0.060. This indicates that the model is a good fit for the data in this population. In contrast, relative fit indices did not reach commonly recommend cut-off values, CFI = 0.860. The basis for comparison for relative fit indices is a model with no associations between variables. It is likely that the modest correlations between items in the BARQC-V (as discussed in the context of internal consistency) account for the modest CFI value.

#### Internal consistency

Using the 5-factor structure, internal consistency of four of the five BARQC-V sub-scales was slightly below the commonly suggested criterion of 0.7 (Rumination α = 0.66, Assertion α = 0.73, Social Support-seeking α = 0.68, Distraction α = 0.62, and Direct Anger-out α = 0.66). There are three reasons why these values should be interpreted with reference to both the low number of items per subscale and the 3-point response format. First, a low number of items substantially increases the item intercorrelations required to achieve the 0.7 criterion [86]. Second, the 3-point response scale introduces more error variance compared to a response scale with more response options. Finally, despite the limitations of 3-point scales, they are often employed in measures intended for self-response from children for pragmatic reasons. For all sub-scales, none of the included items degraded the sub-scale’s alpha coefficient.

### External validity

Table 5 presents evidence of the *concurrent validity* of the BARQC-V. The Rumination coping style showed a large, positive association with stress; a moderate, positive association with depression and anxiety; a moderate, negative association with emotion-focused coping self-efficacy; and a small, negative association with problem-focused coping self-efficacy, social support-seeking coping self-efficacy, and mental well-being. The Direct Anger-out coping style was positively but weakly associated with depression and stress; negatively but weakly associated with emotion-focused and problem-focused coping self-efficacy and mental well-being; and was not associated with social support-seeking coping self-efficacy. The Assertion anger coping style showed a positive, moderate association with problem-focused coping self-efficacy; a positive but weak association with emotion-focused coping self-efficacy, social support-seeking coping self-efficacy, and mental well-being; and a negative, small association with depression. The Social Support-seeking anger coping style was positively but weakly associated with social support-seeking coping self-efficacy, mental well-being, anxiety and stress. The Distraction anger coping style was positively but weakly associated with emotion-focused and problem-focused coping self-efficacy.

**Table 5:** Intercorrelations between the five sub-scales of the BARQC-V and mental health scales

	Factor 1 Rumination	Factor 2 Assertion	Factor 3 Social Support-seeking	Factor 4 Distraction	Factor 5 Direct Anger-out
Factor 1: Rumination	–	–	–	–	–
Factor 2: Assertion	− 0.01	–	–	–	–
Factor 3: Social Support-seeking	0.27**	0.31**	–	–	–
Factor 4: Distraction	0.07*	0.27**	0.20**	–	–
Factor 5: Direct Anger-out	0.34**	− 0.13**	0.08**	0.01	–
CSES-V: Emotion-focused	− 0.38**	0.23**	− 0.05	0.17**	− 0.15**
CSES-V: Problem-focused	− 0.23**	0.36**	0.03	0.11**	− 0.16**
CSES-V: Social Support-seeking	− 0.21**	0.27**	0.19**	0.04	− 0.04
MHCSF-V	− 0.25**	0.28**	0.11**	0.08*	− 0.13**
CESDR-V	0.44**	− 0.07*	0.10**	0.02	0.18**
DASS21D-V	0.42**	− 0.14*	0.06*	− 0.02	0.14**
DASS21A-V	0.43**	− 0.06*	0.13**	0.05	0.18**
DASS21S-V	0.50**	− 0.08*	0.15**	0.03	0.21**

*Note*: CSES-V: Coping Self-efficacy Scale—Vietnamese version; MHCSF-V: Mental Health Continuum Short Form—Vietnamese version; CESDR-V: Centre for Epidemiologic Studies Depression Scale Revised—Vietnamese version; DASS21D-V: Depression Anxiety and Stress Scales 21 items—Depression sub-scale—Vietnamese version; DASS21A-V: Depression Anxiety and Stress Scales 21 items—Anxiety sub-scale—Vietnamese version; DASS21S-V: Depression Anxiety and Stress Scales 21 items—Stress sub-scale—Vietnamese version

^**^ Correlation is significant at the 0.01 level (2-tailed)

^*^ Correlation is significant at the 0.05 level (2-tailed)

## Discussion

This study established partial evidence of the construct validity of the BARQC-V for use by adolescents attending high school in Vietnam. Removing low- and cross-loading items yielded a 5-factor structure that explained 49.32% of the total variance of the BARQC-V, similar to the total variance explained by the 6-factor structure of the BARQ-C (45.7% for the UK sample and 47.7% for the Dutch sample) [3]. Congruent with the findings of Miers and colleagues [3], a 6-factor structure of the BARQC-V did not emerge. All factors in the 5-factor structure demonstrated acceptable internal consistency.

Concerning external validity, three BARQC-V sub-scales predicted both well-being and mental health difficulties. As hypothesised, the Rumination anger coping style was positively associated with depression and distress, and negatively associated with coping self-efficacy and mental well-being, adding weight to the proposition that Rumination is a maladaptive anger coping style [43], at least in Vietnamese adolescents. Similarly, as hypothesised but with weaker associations than the Rumination anger coping style, the Direct Anger-out anger coping style appears to be a maladaptive anger coping style because it was positively associated with depression and distress, and negatively associated with two aspects of coping self-efficacy (emotion-focused and problem-focused) and mental well-being. Also, as hypothesised, Assertion appears to measure an adaptive anger coping style in Vietnamese adolescents as it was positively associated with coping self-efficacy and mental well-being, and negatively associated with depression.

Contrary to expectations, Social Support-seeking and Distraction did not demonstrate the consistent pattern of associations expected of adaptive anger coping styles. It is possible that Social Support-seeking has elements of problem-solving, an adaptive anger coping strategy (i.e., Item 15, “I leave the situation, find someone to listen to my story, and ask for advice”), as well as elements of the maladaptive rumination anger coping strategy used to strengthen beliefs about the angering event or person [71], (i.e., Item 9, “I leave the situation and look for someone who will agree with me”; Item 21, “I think about the problem first and then talk about it with someone”, and Item 27, “I leave the situation and call a friend or family member to tell him/her how I feel”). Concerning the Distraction anger coping style, created by combining the remaining items from the Avoidance and Diffusion sub-scales after excluding low- and cross-loading items, it is possible that the items in this sub-scale require a level of psychological insight more accessible in adults than adolescents to recognise that keeping busy or trying to forget the angering event were an indirect response to anger.

The decision to exclude 15 BARQC-V items in this validation study was based on the identification of low- and cross-loading items detected with exploratory factor analysis. Recall that the 37-item BARQ was developed to detect anger coping strategies used by adults in Canada [44]. The BARQ-C was developed by rewording some BARQ items to better suit children and adolescents, validating it using a sample of British and Dutch children, and excluding 3 items because they did not load on the respective factors [3]. Interestingly, the two items excluded in this study from the BARQC-V Rumination sub-scale because they did not load on the Rumination factor (Item 4, “I try to understand why I got upset”, and Item 10, “I imagine how I could get even with the person who made me angry”) were also excluded in the original BARQ [3], suggesting that these items are not useful for construct validation.

### Strengths, limitations and suggestions for future research

By providing evidence on multiple aspects of the construct validity of the BARQ-C in a large sample of randomly selected Vietnamese adolescents, this study retained items from all original BARQ sub-scales while yielding a pragmatic measure of three anger coping strategies (Rumination, Direct Anger-out, and Assertion) used by adolescents in a non-Western lower-middle income country. This measure can be used to detect the use of maladaptive anger coping so as to intervene to prevent and diminish depression and other mental health difficulties. Interestingly, Vietnamese adolescents’ use of the Direct Anger-out and Assertion anger coping strategies suggests that contrary to the implied assumption that Vietnamese adolescents would suppress their anger to conform with cultural group norms of harmony, self-restraint and affective control [35–38], globalisation may have diminished the difference between adolescents living in Asian countries and those living in other countries [72] and warrants further research. Further, future research should explore the nature of Social Support-seeking and Distraction to determine whether they are adaptive or maladaptive anger coping strategies, and whether they are used in the same manner across cultures. In addition, future research should examine the BARQC-V’s anger coping strategies as combined predictors of mental wellbeing and depression.

A limitation of this study is its exclusive use of self-report measures which may create problems of response bias and common method variance. Future research should consider using additional sources of information (e.g., a coping diary) to strengthen findings. In addition, generalisation of findings is limited to Vietnamese adolescents attending high school in Hanoi. Hence, future research that includes a wide range of children and adolescents from non-Western, lower-middle income countries would provide an opportunity to strengthen the validity of the BARQC-V. Similarly, as the BARQ-C does not appear to have been honed since 2007, and there does not appear to be a gold standard measure of anger coping, future research that includes a wide range of children and adolescents from Western countries would provide an opportunity to strengthen the validity of the BARQ-C. Further, as the validation of the BARQ does not appear to have been strengthened for nearly two decades, and has not been validated across cultures, future research that includes adults from diverse cultures would be worthwhile to create a gold standard measure of anger coping in adults.

## Conclusions

This study provides sound evidence of the structural aspect of construct validity of the BARQC-V when used with adolescents attending high school in the capital of Vietnam, a lower-middle income country in South-East Asia that tends to value family and the group ahead of individual needs. It also provides partial evidence of the external aspect of construct validity of the BARQC-V: the Rumination, Direct Anger-out, and Assertion sub-scales were found to predict well-being and mental health difficulties in this sample and may be useful in school-based mental health promotion programs, and for school counsellors, psychologists, and primary health care providers who work with adolescents in Vietnam to detect maladaptive anger coping so as to intervene to prevent and diminish depression and other forms of psychological distress in this population. Importantly, the BARQC-V provides a validated measure of three anger coping strategies used by adolescents in Vietnam that can be used as a starting point by future research to develop a gold standard measure of anger coping for adults, adolescents and children world-wide.

## Data Availability

The data that support the findings of this study are available from Dr Thach Tran (thach.tran@monash.edu) but restrictions apply to the availability of these data, which were used under license for the current study, and so are not publicly available. Data are however available from the authors upon reasonable request and with permission of Dr Thach Tran.

## Abbreviations

BARQC-V: Behavioral Anger Response Questionnaire for Children in Vietnam
CSES-V: Coping Self-efficacy Scale—Vietnamese version
MHCSF-V: Mental Health Continuum Short Form—Vietnamese version
CESDR-V: Centre for Epidemiologic Studies Depression Scale Revised—Vietnamese version
DASS21D-V: Depression Anxiety and Stress Scales 21 items—Depression sub-scale—Vietnamese version
DASS21A-V: Depression Anxiety and Stress Scales 21 items—Anxiety sub-scale—Vietnamese version
DASS21S-V: Depression Anxiety and Stress Scales 21 items—Stress sub-scale—Vietnamese version
